# Pure rotation in the temporomandibular joint during jaw opening? A digital motion analysis

**DOI:** 10.1038/s41405-024-00213-8

**Published:** 2024-04-18

**Authors:** Bálint Jász, Tamás Balogh, Szilvia Ambrus, Péter Schmidt, Gréta Lilla Bányai, Szandra Körmendi, Máté Jász

**Affiliations:** 1 Semmelweis University, Faculty of Dentistry, Department of Prosthodontics, Budapest, Hungary; 2 Parodont Clinic, Budapest, Hungary; 3 Pázmány Péter Catholic University, Faculty of Information Technology and Bionics, Budapest, Hungary

**Keywords:** Dental anthropology, Temporomandibular disorders, Mandibular muscles

## Abstract

**Objective:**

In the temporomandibular joint two separate movements of rotation and translation occur in two articular spaces during mouth opening. Over the years, the approach has changed considerably, but it is still subject to controversy as to which of these movement is involved in the initial stage of mouth opening. In the present study, the extent of rotation and translation was investigated in the first 5 mm of mouth opening.

**Materials and methods:**

The study was carried out on 46 volunteers. Anamnesis was taken and patients were examined. For the investigation, an ultrasonic digital motion analyser (KaVo Arcus Digma 2) was applied. The measurements were made after calibration to an arbitrary axis. Each participant performed three open-close movements in succession, starting from maximum intercuspal position.

**Results:**

Data were statistically processed using cubic spline interpolation. Linear regression was then used. The resulting line is significantly (*p* < 0.0001) different from the horizontal that represents only rotational movement.

**Conclusion:**

The results show that during mouth opening from maximum intercuspal position, translation is present continuously in addition to rotation. Therefore, it might be time to re-evaluate the principle of a pure rotational approach.

## Introduction

Accurate knowledge of temporomandibular joint (TMJ) movements plays an important role in prosthetic dentistry. In many animal species, there is simple rotational movement of the TMJ along the intercondylar axis during jaw opening. In contrast, the human TMJ has more complex kinematics. If humans had only rotational movement, the anatomical structures between the mandible and the mastoid process would be compressed by the mandible, and only reduced mouth opening could occur due to this physical barrier. This theoretical limitation is most likely one reason that this human joint developed simultaneous rotating and sliding movements [1].

The TMJ is a diarthrosis that is capable of rotating along multiple axes. These free movements are limited by chewing muscles, tooth contact, articular ligaments and capsule. The articular disc separates the joint into two articular compartments. Translational movements occur in the cranial discotemporal joint, while rotational movements take place in the caudally located discomandibular joint. Rotation and translation occur simultaneously during mouth opening, resulting in complex movements.

According to the hinge axis theory, the transverse or hinge axis of the mandible is an imaginary line around which the mandible rotates in the sagittal plane at the initiation of opening movements and at the end of closing movements. Several methods to determine the position of this axis have been developed and described in the literature [2–4]. This theory has gained fundamental importance for dental interventions, including prosthodontic rehabilitations. With mean-value and semiadjustable articulators, if the vertical dimension increases or decreases within a certain limit in the articulator, i.e., in the case of a small change in the OVD, we assume that the position of the condyle in the articular fossa remains unchanged [2]. Scientific research investigating the stomatognathic system has mostly been based on pure rotational movement, as this is a widely accepted theory [5–9]. The principle of purely rotational movement plays an important role not only in prosthodontics but also in orthodontics and orthognathic surgery [10, 11].

Prosthodontic appliances made in articulators with improperly modelled jaw movements might lead to several adverse consequences, such as occlusal disturbances and incorrect articulation tooth wear, periodontal overload, loosening of removable dentures, disturbance of the temporomandibular joint, and discomfort [12].

In the 1960s, the range of pure rotation was determined up to a mouth opening of 15–20 mm [13]. The extent of this range has changed with the development of different gnathological examination procedures. According to the latest research, pure rotational movement likely does not occur [14, 15]. Therefore, overall, an extremely crucial question in dentistry is whether there is a purely rotational movement in the temporomandibular joint.

The present study investigated whether and to what extent there is a pure rotational movement in the temporomandibular joint during the initial phase of mouth opening. The degree of translation in relation to the degree of opening was examined in the first 5 mm of the jaw opening, as the literature currently defines the range of possible pure rotational movement within this interval [16, 17]. The null hypothesis was that pure rotational mouth opening does not occur.

## Materials and methods

The study was conducted with the participation of 46 volunteers. The inclusion criteria for the study were as follows: (1) good general health and absence of developmental disturbances related to the temporomandibular jaws or the teeth; (2) preserved or restored teeth (excluding third molars); (3) restored dentition, with no guiding surfaces of the restoration during articulatory movements; (4) no orthodontic treatment in the anamnesis; (5) no history of temporomandibular disorders (TMD) and no present TMD signs or symptoms; and (6) no possible or probable awake bruxism or sleep bruxism, no parafunctions based on the patient’s history and intraoral examination, and no abnormal tooth guidance (e.g., hyperbalances or premature contact); and (7) straight opening pattern.

Our study received ethical approval from the Regional, Institutional Scientific and Research Ethics Committee (No. 92/2013). The study was conducted between 2014 and 2020. All participants were informed about this study and signed an informed consent form.

A KaVo Arcus Digma 2 digital motion analyser (KaVo Gmbh, Biberach, Germany) was used for this investigation. This device operates by conducting measurements based on ultrasound signals and comprises two primary components. The lower unit is affixed to the vestibular surface of the lower teeth using a cold polymerising acrylate material (Structur II, Voco GmbH, Cuxhaven, Germany) connected to a paraocclusal clutch, which, in turn, links to four transmitters through magnets. Throughout the measurement process, this unit remained securely attached to the mandible. The upper unit is fastened to a facebow, with the Frankfort horizontal plane serving as the reference plane.

Ultrasound signals, emitted in the form of regular cones, are generated by the transmitters. Each transmitter corresponds to four detectors, resulting in a continuous connection comprising 16 signal pairs. This digital motion analyser completes a measurement in two hundredths of a second, so fifty registrations occur in each second. The measurements were carried out using the manufacturer’s own software (KaVo KiD, KaVo GmbH, Biberach, Germany). Each measurement was performed by the same trained examiner. Two randomly selected patients were re-examined. The second registrations were duplicates and were not included in the final statistical analysis. The Weighted Kappa Coefficients ranged from 0.87 to 0.92 for intraexaminer reliability.

At the beginning of the measurement, the calibration was performed in “arbitrary axis” mode. In this case, the instrument was calibrated to the Frankfort horizontal plane before the measurement was started. That means, the calibration was performed to the lateral poles of the condyles and to the infraorbital point using a calibration pin. The position of the lateral pole of the condyle was identified by palpation during multiple mouth openings. The position of the condyle relative to the maximum intercuspal position (MIP) was then marked with a pen. The point thus marked was used for calibration. The test was performed in motion analysis mode of the device. In all cases, the starting position of the movement was the MIP. From there, the patients performed opening‒closing movements three times in a row. The manufacturer’s software was used to calculate the average of the three openings, and the data were exported. (KaVo KiD, KaVo Gmbh, Biberach, Germany). For further data analysis, since the sampling was done at a given time-frequency and not at a given mouth opening positions cubic spline interpolation was used to re-sample the data to have data for each given value from each measurement, thus allowing for averaging and statistical evaluation of the measurements. Cubic spline interpolation, without any additional smoothing, was computed using MATLAB’s spline() function and statistical analysis was performed using GraphPad Prism (GraphPad Software, Boston, USA) statistical software.

## Results

**Fig. 1:**
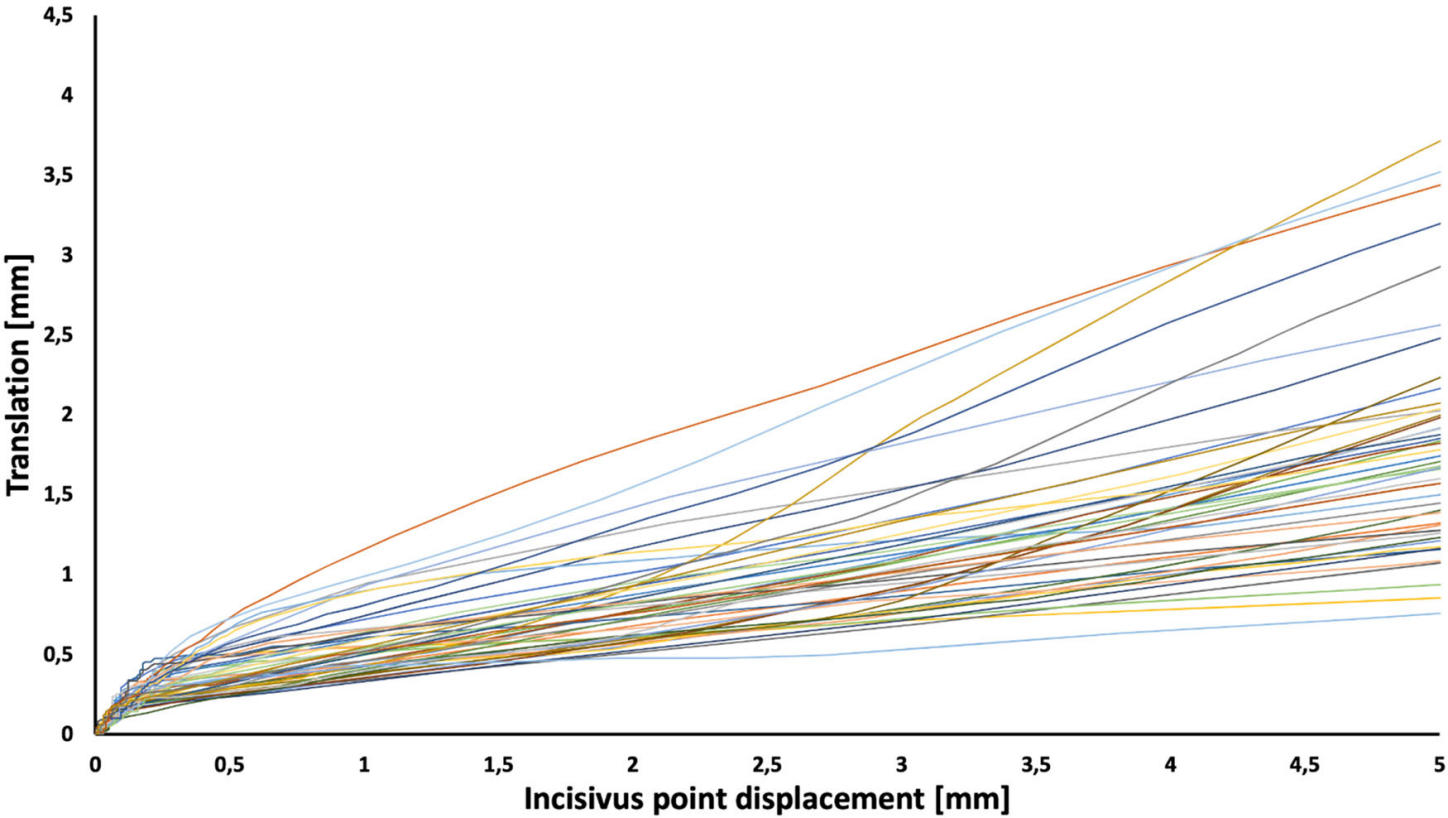
Translational movement of the condyle as a function of the displacement of the incisivus point. The x-axis shows the displacement of the incisivus point in mm, while the y-axis presents the translation in mm for each patient indicated by different colours.

**Fig. 2:**
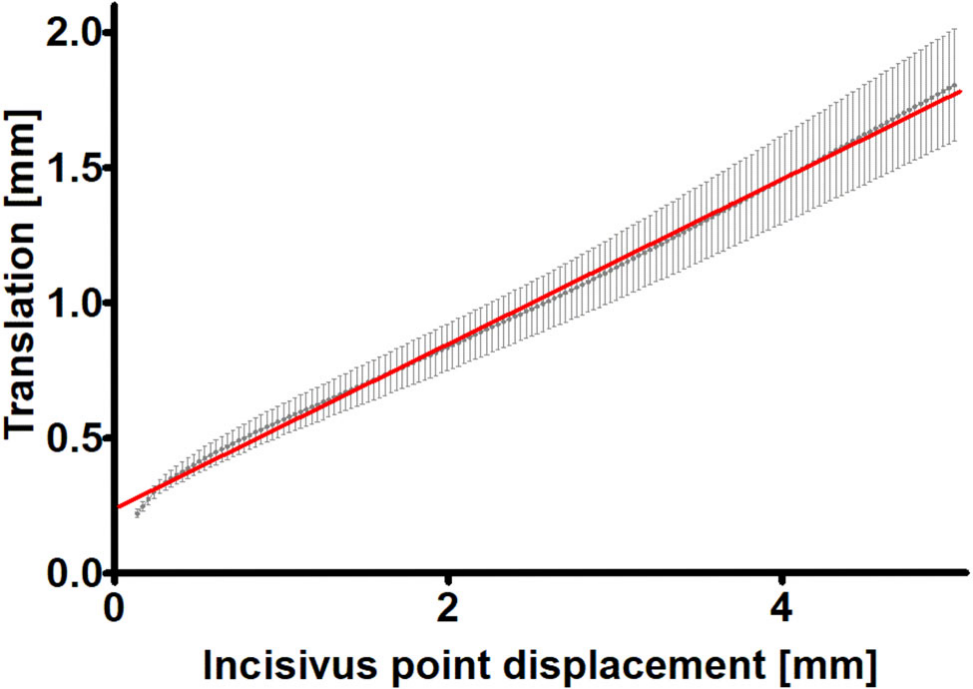
Displacement of the incisivus point (mm) as a function of the translational movement of the condyle (mm) and the best-fitting regression line. The data are shown as the average ± 95% CI; *n* = 46.

During the examination, the software recorded three-dimensional coordinates of the three Bonwill points every two hundredths of a second. From the KaVo KiD, it was possible to export the coordinates for each time point. From these coordinates, it was then possible to mathematically determine the followed paths in millimetres to two decimal places [18]. This allowed visualisation of the quantity of translational movements at each stage of mouth opening for each patient. The translation of the condyles was plotted as a function of mouth opening for each individual examined (Fig. 1). The data obtained at the initial 0.2 mm of opening (initial, steeper section in Fig. 1) were not considered in the evaluation of the results. These sections were most likely artificial due to the physical size of the transducers on the paraocclusal clutch of the instrument and its inertia at the start of the movement. After the statistical analysis was conducted, the gathered data were transformed into continuous variables. Through repeated sampling, we quantified the relationship between translation and opening using linear regression, as shown in Fig. 2. The best-fitting regression line yielded an impressive coefficient of determination (R^2^) of 0.9981. It had a slope of 0.3044 (with a 95% confidence interval of 0.3023 to 0.3065) and an intercept of 0.2382 (with a 95% confidence interval of 0.2318–0.2445). Notably, the calculated slope was found to be significantly different (*p* < 0.0001) from a slope of 0, which corresponds to a pure rotation in relation to the arbitrary hinge axis, essentially representing a horizontal line.

## Discussion

With a digital motion analyser, the condylar path during normal mandibular movements can be tracked much more accurately than with previous mechanical motion analysers [5]. Digital motion analysers can also support the process of diagnosing intracapsular pathologies of TMD. In certain TMJ pathologies, characteristic movement trajectories can be observed, which is an important aid in differential diagnosis. Additionally, with the data obtained from the digital motion analyser, it is possible to determine individual movement trajectories for the programming of semiadjustable or fully adjustable articulators [5, 19].

**Fig. 3:**
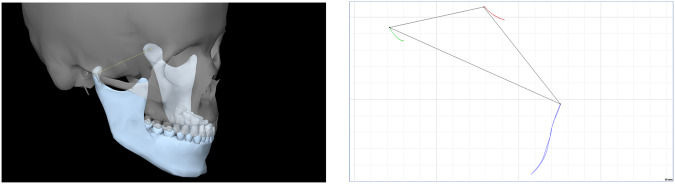
Left: Virtual representation of the mandible and the intercondylar axis connecting the condyles. Right: Trajectories of the Bonwill points during mouth opening and closing and the Bonwill triangle. (KaVo KiD, KaVo Gmbh, Biberach, Germany).

**Fig. 4:**
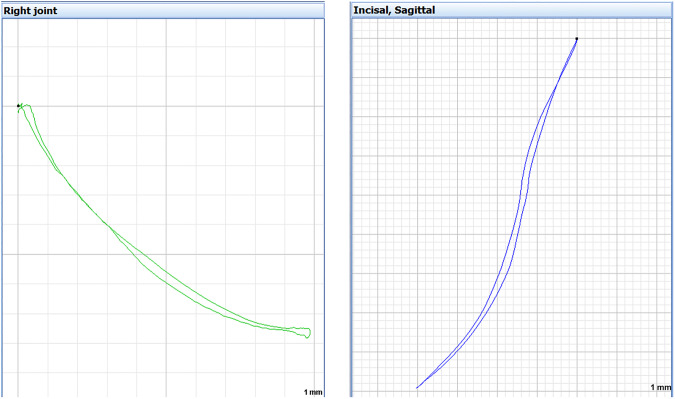
Left: Right condylar path during mouth opening and closing in the sagittal plane, view from the right side: it slides forwards and downwards on the anterior slope of the glenoid fossa to maximum mouth opening and then returns to its original position while closing. Right: Trajectory of the incisivus point during opening and closing of the mouth, right view, sagittal plane (KaVo KiD, KaVo Gmbh, Biberach, Germany).

After digital data processing, the software displays the displacements of the three Bonwill points in an arbitrary plane, which was the Frankfort horizontal plane in our examination (Figs. 3 and 4). Kinematic facebows allow the true axis of rotation to be determined with greater accuracy but with complexity. These devices, due to their complexity, have not spread but have been superseded by artbitrary facebows. The use of ar arbitrary facebows represents a minimal compromise in the accurate determination of the axis of rotation. Based on dental practice over the past few decades, this discrepancy does not represent a clinically relevant source of error. The system allows motion analysis with an accuracy of one hundredth of a millimetre according to the manufacturer’s data [18]. From the results obtained, it is clear that translation is continuously present during mouth opening from the first mm of the opening starting from the MIP. This contradicts the previously described finding that pure rotational movement occurs in the first 10–20 mm of mouth opening [2, 4].

For almost a century, gnathologists have been working to understand the individual movement pathways of the mandible. Since the beginning, the presence and amount of rotational movement in the first half of the opening has been a matter of discussion. First, the concept of rotational (hinge) movement around the intercondylar axis was dominant. Techniques for determining the position of the true individual axis of rotation, i.e., the terminal hinge axis (THA), were developed in the 1960s by McCollum and Lucia [2, 3]. The recording device they used is called the kinematic pantograph. The principle of its operation is that a drawing device attached to the mandible draws regular circles on the registration surface attached to the maxilla/skull during the opening‒closing movement in cases of rotation. By changing the position of the drawing device, a position where only one point appears during opening and closing can be achieved. This indicates the presence of pure rotational movement, and the tip of the drawing tool then points to the THA. If no point can be detected, this indicates the presence of a translation. To date, digital versions of the kinematic pantograph are still available in dental practice and for scientific studies [17].

A study conducted in 2018 attempted to determine the ability of the pantograph to distinguish between pure rotation and combined rotational and translational movement. The researchers also analysed the degree of inaccuracy in the determination of THA caused by the presence of a translational component in the condylar path. The analysis was mathematically derived, and the results were verified by computer simulation. The results showed that these instruments are not suitable for detecting pure rotation: a translation of 1.1 mm can result in THA displacement of 6.7 mm, and a translation of 2.2 mm can result in THA displacement of up to 13.5 mm [20]. All these outcomes indicate that the results from published mechanical pantograph studies used to determine THA should be interpreted with caution.

Jaw movements have also been investigated using other methods [21]. A method to determine the instantaneous centre of rotation (ICR) was described in the 1970s. Experience in the field of anatomy has shown that bones rarely move along only one axis. In 1973, Grant described a model based on this theory [22]. The authors’ mathematical deduction was that the direction of tension of the muscles explains the translation along a constantly changing axis during mouth opening and closing, and thus, based on the results, they rejected the possibility of pure rotational movement. Keeping Grant’s approach but examining and calculating the ICR using a different method, Chen also concluded that the ICR changes continuously during opening and closing, so it cannot be considered pure rotation. In this in vivo study, the current position of the ICR was calculated from positional changes in four marker points based on photographs of seven individuals. However, the ICRs are closer to the centre of the condyles during the initial stage of opening (a mouth opening of 10 degrees), indicating the dominance of rotation over sliding movement. A limitation of their study is that the method allowed only two-dimensional investigation [23]. Ferrario et al. used a kinesiograph (Sirognathograph) to investigate the location of the ICR and the resulting instantaneous centre of curvature (ICC) during opening and closing. Their results showed that pure rotational movement was not present during either opening or closing; thus, the ICR theory was proven to be correct. Furthermore, they found that the speed of movement affected the position of the ICR [1].

Ahn et al. used a digital model to investigate changes in the ICR position during opening and closing of the mouth with a pantograph in virtual space. In a highly accurate study with a resolution of one thousandth of a second (0.001 s), the ICR did not have a constant position. The points recorded during opening and closing did not coincide with each other, presumably as a result of different muscles acting on the mandible during opening and closing movements. A distance of less than 1 mm was detected between the instantaneous centre of rotation measured at the starting position and at the 10 mm mouth opening. Considering the perceptual limitations of the human eye and the inaccuracy of previous mechanical measuring instruments, the earlier results of pure rotation can be explained [24]. This result is less than the amount/degree of translation recorded in this study, which was close to 2 mm for the first 5 mm of the mouth opening. The reason for the difference between the results of the two studies is not entirely clear but may be related to the fact that some researchers have questioned the relevance of the ICR, as its measurement accuracy raises several problems, and it has been found to be insufficiently accurate and reproducible [25].

In an in vivo study, Mapelli et al. investigated the relative contribution of rotation and translation to mandibular movements during mouth opening and mouth closing using an optoelectronic instrument that provides 3D images. For each patient, the shifts in the incisivus point and the condyle points were recorded. In addition, the degree of rotation of the mandible around the intercondylar axis was measured. To compare the results, the trajectory of the sagittal projection of the incisivus point to maximum mouth opening was divided into ten equal parts. For each section, the incisivus point displacement attributable to rotation was examined. The amount of rotation was more prevalent than the amount of translation in each section but never approached 100%; consequently, no pure rotation was found. The extent of translation was similar between sexes, but males had longer condylar paths than females regardless of the mandibular size (*P* < 0.05). A linear correlation between maximum mouth opening and the condylar path during translation was also examined, but no significant association was found [16].

Based on the results of the present study, a new test method could demonstrate that there is no pure rotation in relation to the arbitrary hinge axis in the temporomandibular joint at the initial stage of mouth opening. Additionally, translation is present from the beginning of movement. This finding is consistent with the most recent research in the literature.

## Conclusion

The minimal difference in distance between the arbitrary axis and the kinematic axis was used in the present study. This is a clinically relevant finding as the arbitrary axis is the basis of a number of clinical concepts. Considering similar findings in the literature, the continued applicability of the pure rotational approach should be reconsidered.

## Data Availability

The datasets used and analysed during the current study are available from the corresponding author upon reasonable request.

## References

[1] Ferrario VF, Sforza C, Miani A, Serrao G, Tartaglia G (1996). Open-close movements in the human temporomandibular joint: does a pure rotation around the intercondylar hinge axis exist?. J Oral Rehabil.

[2] McCollum BB (1960). The mandibular hinge axis and a method of locating it. J Prosthet Dent.

[3] Lucia VO (1960). Centric relation—Theory and practice. J Prosthet Dent.

[4] Posselt U (1957). Terminal hinge movement of the mandible. J Prosthet Dent.

[5] Piehslinger E, Celar AG, Celar RM, Slavicek R (1991). Computerized axiography: principles and methods. Cranio.

[6] Ishigaki S, Nakamura T, Akanishi M, Maruyama T (1989). Clinical classification of maximal opening and closing movements. Int J Prosthodont.

[7] Nagy WW, Smithy TJ, Wirth CG (2002). Accuracy of a predetermined transverse horizontal mandibular axis point. J Prosthet Dent.

[8] Roth RH, Williams RE (1996). Comment on condylar movement and mandibular rotation during jaw opening. Am J Orthod Dentofac Orthop.

[9] Stern N, Hatano Y, Kolling JN, Clayton JA (1988). A graphic comparison of mandibular border movements generated by various articulators. Part I: Methodology. J Prosthet Dent.

[10] Lindauer SJ, Sabol G, Isaacson RJ, Davidovitch M (1995). Condylar movement and mandibular rotation during jaw opening. Am J Orthod Dentofac Orthop.

[11] Liebregts J, Baan F, van Lierop P, de Koning M, Bergé S, Maal T (2019). One-year postoperative skeletal stability of 3D planned bimaxillary osteotomies: maxilla-first versus mandible-first surgery. Sci Rep.

[12] Farook TH, Rashid F, Alam MK, Dudley J (2023). Variables influencing the device-dependent approaches in digitally analysing jaw movement-a systematic review. Clin Oral Investig.

[13] Hickey JC, Boucher CO, Hughes GA (1968). Glossary of prosthodontic terms. J Prosthet Dent.

[14] Villamil MB, Nedel LP, Freitas CM, Macq B (2012). Simulation of the human TMJ behavior based on interdependent joints topology. Comput Methods Prog Biomed.

[15] Hugger A, Hugger S, Ruge S, John D, Kordaß B (2020). The rotation vs translation behavior during habitual opening and closing movements of the mandible and the relationship to movement paths of condylar points. Int J Comput Dent.

[16] Mapelli A, Galante D, Lovecchio N, Sforza C, Ferrario VF (2009). Translation and rotation movements of the mandible during mouth opening and closing. Clin Anat.

[17] Mehl A (2020). Is it possible to detect a true rotation axis of the temporomandibular joint with common pantographic methods? A fundamental kinematic analysis. Comput Methods Biomech Biomed Engin.

[18] KaVoDental. Arcus Digma Instructions for Use 2008. 2023, https://kavo.widen.net/content/5f6sxpksek/original/GA_ARCUSdigmaII_20080619_01_en.pdf?u=ai5cab&download=true.

[19] Stiesch-Scholz M, Demling A, Rossbach A (2006). Reproducibility of jaw movements in patients with craniomandibular disorders. J Oral Rehabil.

[20] Mehl A (2018). Hinge axis determination of the temporomandibular joint and its interpretation: what do we really measure?. Int J Comput Dent.

[21] Gallo LM, Airoldi GB, Airoldi RL, Palla S (1997). Description of mandibular finite helical axis pathways in asymptomatic subjects. J Dent Res.

[22] Grant PG (1973). Biomechanical significance of the instantaneous center of rotation: The human temporomandibular joint. J Biomech.

[23] Chen X (1998). The instantaneous center of rotation during human jaw opening and its significance in interpreting the functional meaning of condylar translation. Am J Phys Anthropol.

[24] Ahn SJ, Tsou L, Antonio Sánchez C, Fels S, Kwon HB (2015). Analyzing center of rotation during opening and closing movements of the mandible using computer simulations. J Biomech.

[25] Chen J, Katona TR (1999). The limitations of the instantaneous centre of rotation in joint research. J Oral Rehabil.

